# Removal of amoxicillin from contaminated water using modified bentonite as a reactive material

**DOI:** 10.1016/j.heliyon.2024.e24916

**Published:** 2024-01-22

**Authors:** Alaa K. Mohammed, Sara M. Saadoon, Ziad T. Abd Ali, Israa M. Rashid, Nadya Hussin AL Sbani

**Affiliations:** a Biochemical Engineering Department, Al-Khwarizmi College of Engineering, University of Baghdad, Baghdad, 47024, Iraq; b Environment Eng. Dept. / College of Engineering / University of Baghdad, Baghdad, 47024, Iraq; c Department of Chemical Engineering, Al Zawia University, Libya

**Keywords:** Modified-bentonite, Amoxicillin, Kinetics, Isotherms, Thermodynamics

## Abstract

This study concerns the removal of a trihydrate antibiotic (Amoxicillin) from synthetically contaminated water by adsorption on modified bentonite. The bentonite was modified using hexadecyl trimethyl ammonium bromide (HTAB), which turned it from a hydrophilic to a hydrophobic material. The effects of different parameters were studied in batch experiments. These parameters were contact time, solution pH, agitation speed, initial concentration (C_0_) of the contaminant, and adsorbent dosage. Maximum removal of amoxicillin (93 %) was achieved at contact time = 240 min, pH = 10, agitation speed = 200 rpm, initial concentration = 30 ppm, and adsorbent dosage = 3 g bentonite per 1L of pollutant solution. The characterization of the adsorbent, modified bentonite, was accomplished using Fourier transform infrared spectroscopy, scanning electron microscopy, X-ray diffraction, and Brunauer-Emmett-Teller. The isotherm models were also investigated, and it was found that the Freundlich isotherm model fitted well with the experimental data (R^2^ = 94.77), which suggests heterogeneity in the multilayer adsorption of amoxicillin onto modified bentonite. The kinetics of the adsorption process were studied. The experimental data were found to obey the pseudo-first-order kinetic model (R^2^ = 95.1). Thermodynamic studies indicated that the adsorption process was physisorption and endothermic. Finally, the modified bentonite proved to be a good adsorbent for the removal of amoxicillin from contaminated solutions.

## 1 Introduction

Pharmaceutical products were considered the most important environmental pollutants [1,2]. In general, pharmaceuticals are substances used in the treatment, diagnosis, and prevention of disease. They are used for the promotion of human health, animal treatment, and agriculture [3]. Antibiotics are the most widely used pharmaceuticals to treat bacterial infections in humans, animals, and plants [4]. Amoxicillin (AMX) is the most popular antibiotic due to its resistance against a wide spectrum of microorganisms. AMX has two fundamental parts, which are the β-lactam inner and the side chain known as Dhidroxiphenilglicin [5]. AMX residue hurts human health and the environment. It causes skin complaints and a spiteful odor. Also, it may cause losses of microorganisms that are operative in wastewater treatment or cause bacterial resistance among pathogen organisms [6]. Resistant bacteria may cause diseases that are difficult to treat using conventional antibiotics [7]. Therefore, the removal of AMX residue is getting great attention. The techniques for removing AMX waste are quite expensive. The medicinal industries must treat their waste before discharging it into the environment [8]. Several methods have been experienced for AMX waste treatment, such as electrochemical degradation [9], ozonation [10], advanced oxidation [11], electrocoagulation [12], nanofiltration [13], photocatalytic degradation [14], and adsorption processes [15,16]. The adsorption method is more efficient and restricts the pollutant's transportation into the water system [15].

In adsorption processes, different adsorbents have been used for the removal of contaminants or organic compounds such as dyes, antibiotics, and heavy metals from industrial, municipal, groundwater, and drinking water [17,18]. One of the most commonly used materials as adsorbents for amoxicillin removal is activated carbon [19]. However, using activated carbon as an adsorbent may face many challenges; one of them is the high cost of preparation [20,21]. Bentonite is an inexpensive, readily available material that has been widely used in material science technology, so in recent years, great attention has been paid to using this clay material as an adsorbent for the removal of hazardous substances [22]. In contrast to activated carbon, where adsorption occurs within the pores, this action occurs on the surface of the clay platelets. When a large organic molecule passes through activated carbon, it either fills or blinds the pore. Because of this, modified clays are good at absorbing soluble organic molecules from watery solutions that aren't very strong [23]. The clay and the cationic tailoring agents have a strong electrostatic attraction. The charge differences between the negative charge of bentonite and the positive charge in the quaternary amine group cause this electrostatic interaction [24]. Table 1 summarizes the previous works that utilized various modified minerals, such as clay, for the adsorption of several contaminants from polluted water in the batch-mode process. For a long time, the pharmaceutical industry has been concerned about the interaction between clay minerals and antibiotics [[25], [26], [27]]. Adding surfactants to bentonite changes it smoothly. Surfactants are molecules with a quaternary ammonium group that attracts water and an alkyl chain that repels water, like hexadecyl trimethyl ammonium bromide [28]. In the modification process, the inorganic cations are replaced by organic cations, which results in changing the surface properties of the clay from highly hydrophilic to progressively hydrophobic, hence improving the adsorption capacities of the clay [23,26]. Recent studies reported that modification of bentonite with hexadecyl trimethyl ammonium bromide (HTAB) improves the removal of antibiotics from aqueous solutions [29,30]. Adding HTAB to the natural bentonite will enhance the efficacy of removing amoxicillin. This behavior is due to the electrostatic attraction force between the anionic molecules of the pollutants and the positive charge of the modified bentonite surface [31]. Furthermore, the spacing between the bentonite layers expanded during the modification process, resulting in the transformation of the bentonite's hydrophilic surface into a hydrophobic one. This modification was carried out to enhance the adsorption capabilities of the bentonite. This study can be utilized to treat wastewater near pharmaceutical industries as well as wastewater in the vicinity of hospitals and medical centers. The water may contain several antibiotics at doses exceeding the permissible limits. Hence, bentonite can be employed to eliminate antibiotic pollutants from contaminated water. This study aims to investigate the effects of experimental conditions such as contact time, acidity pH, initial concentration of pollutants, mixing rate, and dosage of adsorbent on the adsorption of amoxicillin using modified bentonite with cationic surfactant (HTAB). In the present study, Akaike's information criterion (AIC) was also applied.

**Table 1:** Previous works summary of some contaminants adsorption in batch mode using various clay mineral.

Clay mineral	Organic contaminant type	Type of modification	Adsorption efficiency or capacity	reference
Bentonite	diclofenac (DCF) and cadmium (II)	organobentonite was synthesized by adsorbing the surfactant hexadecyltrimethylammonium (HDTMA)	The sorption capacity of OBHDTMA for DCF was influenced by the solution pH, ionic strength, and temperature	[28]
Bentonite	Amoxicillin	hexadecyl trimethyl ammonium (DK1)	the removal showed 81.9 % and 87.5 % at 19 ppm and 2 ppm, respectively	[29]
Bentonite	Diclofenac sodium	the surfactant hexadecyltrimethylammonium (HDTMA)	The maximum sorption capacity of the OBHDTMA for DCF was 388 mg/g	[32]
Bentonite	Synthetic Estrogen	Trp-Na-bent and Fe–Na-bent were produced by modifying them with L-Tryptophan and FeCl2·4H2O, respectively.	The maximum adsorption capacity was 4.20 mg/g.	[33]
Bentonite	Amoxicillin (AMX)	HTAB	93 %	–

## 2 Materials and methods

### 2.1 Materials

Adsorbent:

Iraqi bentonite was used as the adsorbent in this investigation; the rock fragments were provided by the State Company of Geological Survey and Mining (Baghdad). The State Company of Geological Survey and Mining's laboratories were used to examine the physical properties and content of Iraqi bentonite. In Table 2, the composition is displayed. Hexadecyl trimethyl ammonium bromide was the surfactant used to modify natural bentonite (HTAB). This surfactant was utilized without further purification and was of analytical grade.

**Table 2:** Characteristics of Iraqi bentonite (State Company of Geological Survey and Mining, Petroleum Research and Development Center).

Component	Composition wt%
SiO_2_	50.05
Al_2_O_3_	16
CaO	7.74
FeO_3_	6.26
MgO	3.14
Na_2_O	1.01
LO/1	11.44

**Fig. 1:**
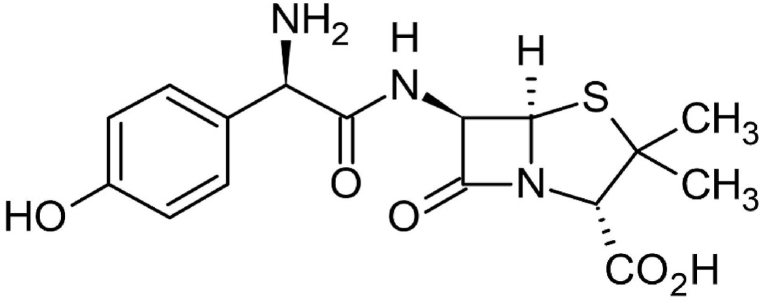
Chemical structure of Amoxicillin.

**Adsorbate:** Amoxicillin was supplied by the State Enterprise for Drug Industries and Medical Appliances, Samara, Iraq. This material was utilized in its raw form without undergoing any additional processing. Amoxicillin's structure displays amphoteric capabilities as a result of three primary functional groups: NH_2_, COOH, and OH, with the chemical formula C_16_H_19_N_3_O_5_S, as shown in Fig. 1 [34]. Amoxicillin has three different acid dissociation values because of its diversity: pKa 1 = 2.68 (carboxyl group), pKa 2 = 7.49 (amine group), and pKa 3 = 9.63 (phenol group) [35]. Amoxicillin can be present in an aqueous solution in four distinct forms (AMX^+^, AMX, AMX^−1^, and AMX^−2^) based on the pH of the solution.

### 2.2 Preparation of modified bentonite

**Fig. 2:**
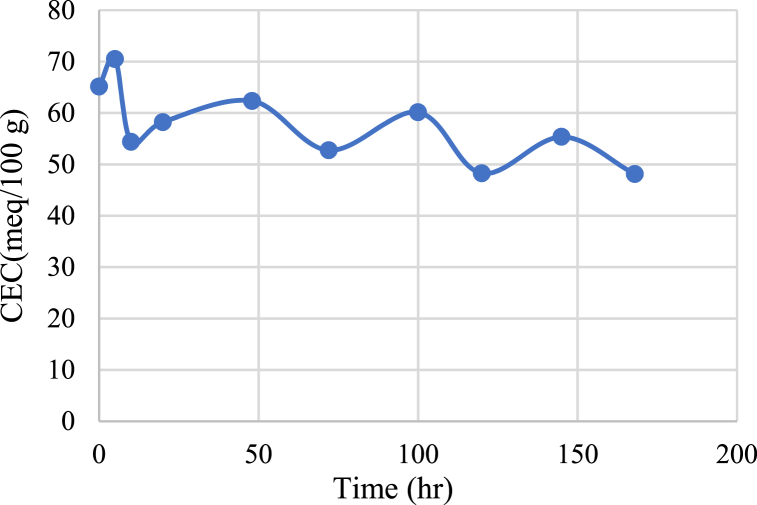
CEC versus time of alteration.

The procedure for the organo-bentonite preparation is illustrated as follows: (i) Dispersing 10 g of natural bentonite in 200 ml of distilled water with agitation at 200 rpm for 2 h. (ii) Adding 0.37 g of HTAB to the mixture and still mixing at 200 rpm for an additional 2 h (iii) The suspension was filtered using filter paper (Whatman No. 40) to separate the bentonite from the aqueous solution and then washed with distilled water several times to remove excess salts. (iv) Finally, the mixture was dried at 100 °C for 6 h. The dried material was then ground using mortar and sieved with a size range of 355–710 μm. The value of the cation exchange capacity CEC of bentonite was determined using NaOH (1 M) according to the method used by Calabria et al. [36]. The values of CEC ranged from 48.12 to 65.14, as shown in Fig. 2.

#### 2.2.1 Adsorption experiment

Batch experiments were performed using 100 ml of the synthetic solution of amoxicillin (50 mg/L) added to an Erlenmeyer flask (250 ml), and then a known amount of modified bentonite (MB) was added to the solution. The solution was agitated using an orbital shaker (Edmund Buhler SM25, German) at a speed of 200 rpm for 4 h. Subsequently, the suspension underwent filtration using Whatman filter paper to remove the adsorbent from the aqueous solution. The AMX's final concentration was analyzed using a double-beam UV–visible spectrophotometer (PG Instruments, Model UV T80, England). The experiment was replicated three times to ensure the obtained results. The wavelength corresponding to the AMX compound was determined to be λ = 228. Equation (1) was employed to compute the percentage of AMX removed by MB [37,38].

(1) $$R=\left[\frac{C₀-C_{e}}{C₀}\right]*100\%$$

R: represents the removal percentage, C₀: is the amoxicillin initial concentration (mg/L), and Ce: is the equilibrium concentration (mg/L). The equilibrium adsorption capacity of bentonite for AMX, qₑ (mg/g), was calculated using Eq. (2) [39,40]

(2) $$qₑ=\frac{\left(C₀-C_{e}\right)V}{m}$$

Where qₑ: AMX adsorbed amount (mg/g); V= the volume of the sample (ml) and m is the quantity of the adsorbent added (g).

### 2.3 Characterization of activated carbon

The morphology of the organobentonite was examined using a Scanning Electron Microscope (SEM) examination. The FTIR 8400s Shimadzu was employed to analyze the functional group present on the surface of the adsorbent, as well as to examine the properties of both the natural and modified bentonite. This analysis was conducted using KBr pellets within the frequency range of 4000–400 cm^−1^. X-ray diffraction (XRD) studies were performed using a Shimadzu XRD 6000, an X-ray diffractometer from Japan. The Cu radiation target is scanned continuously from Theta −2, with a scan speed of 5.0000 deg/min and a preset time of 0.60 s. The surface area analysis was conducted using a BET surface area analyzer (BET: HORIBA, SA-900 series, USA). The catalyst sample undergoes an initial degassing process in a vacuum and at a constant temperature to remove any physisorbed volatile substances and contaminants that may have been absorbed from the surrounding atmosphere. Subsequently, a cryogenic nitrogen gas (N_2_) at a temperature of 77 K passes the solid surface of the catalyst using a volumetric flow method at a specific pressure. The gas particles adhere to the catalyst surface of an undefined shape, resulting in the formation of a monomolecular layer. The gas molecules will have a limited duration of time on the surface, and the quantity of gas that is adsorbed is connected to the provided pressure, enabling us to determine the surface area of the catalyst particles.

## 3 Results and discussion

### 3.1 Characterization using FT-IR

**Fig. 3:**
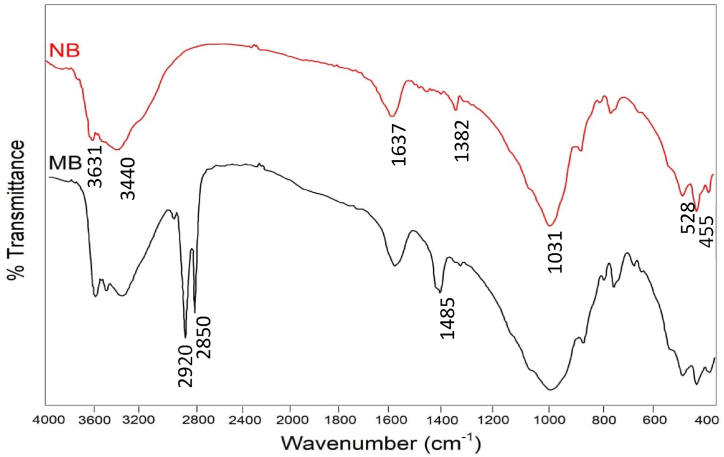
FTIR spectra of natural bentonite (NB), and modified bentonite by HTAB (MB).

The FTIR spectra of both natural bentonite (NB) and modified bentonite (MB) are shown in Fig. 3. The peak at wave number 3631 cm^−1^ refers to the stretching vibration of O–H in the Si–OH and Al–OH groups of the montmorillonite. A broad peak at wave number 3440 cm^−1^ is assigned to the O–H stretching vibrations, while the peak at 1637 cm^−1^ is attributed to the O–H bending vibrations. The peak at wave number 1382 cm^−1^ corresponds to the CO_3_ stretching of the calcite. The in-plan stretching vibrations of the Si–O group were observed at wave number 1031 cm^−1^. The Si–*O*–Si and Al–*O*–Al bending vibrations appear at 455 cm^−1^ and 528 cm^−1^, respectively. For the MB, the stretching vibrations of the CH_3_ and CH_2_ groups of the HTAB appear at 2920 cm^−1^ and 2850 cm^−1^ respectively. The wave number 1485 cm^−1^ corresponds to the flexural vibration of C–C–C of the methylene group that is associated with the hexadecyl trimethyl ammonium ion. This confirms the success of the modification of montmorillonite [41].

### 3.2 SEM analysis

**Fig. 4:**
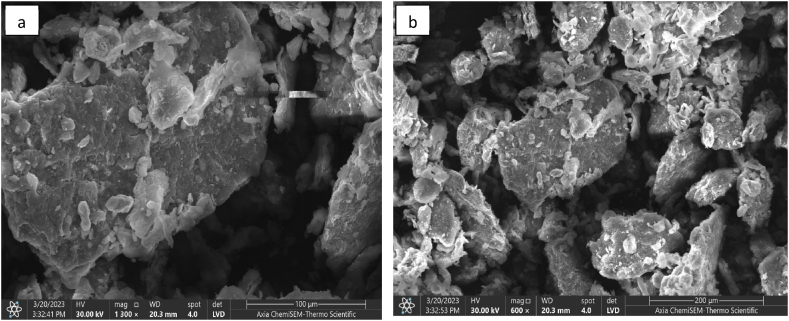
SEM images of (a) Natural bentonite (b) Modified bentonite (MB).

The microstructure and surface morphology of the natural bentonite were examined by SEM analysis, both before and after treatment with HTAB (MB), as depicted in Fig. 4. Observation revealed notable alterations in the surface morphology of the original bentonite following its treatment with HTAB. It was observed that the surface morphology of natural bentonite (NB) is very smooth, massive, aggregated, and fluffy, while the modified bentonite has a rough surface. This is because the natural bentonite loses some of its foliated structure during the insertion reaction of the cationic surfactant. Also, deformed parts were shown in the micrograph of the natural bentonite, which may have occurred due to the reduction in certain crystalline domains of the bentonite particles. The basal spacing of modified bentonite increased from 36.66 nm to 53.54 nm [42].

### 3.3 XRD analysis

**Fig. 5:**
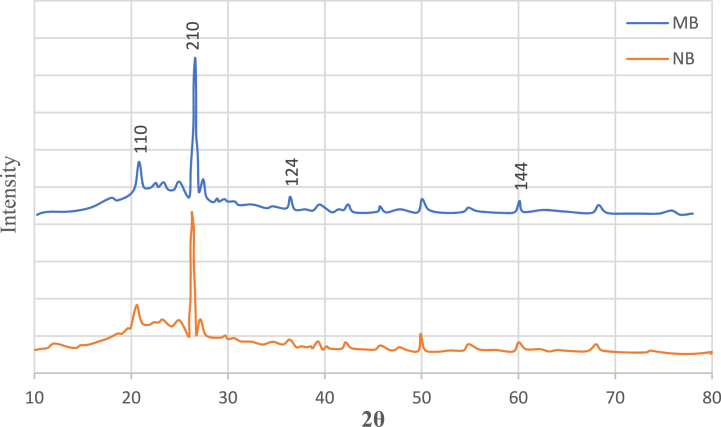
The XRD analysis of natural bentonite, (NB) and modifed bentonite. (MB).

Fig. 5 displays the XRD patterns for both modified and natural bentonite nanoparticles. At diffraction angles 2θ = 20.9°, 26.3°, 36.6°, and 54.5°, the distinctive peaks correspond to the bentonite material's planes (110), (210), (124), and (144). These XRD patterns match the standard JCPDS file (card no. 01-088-0891) rather well. It is evident from the graph that neither the peaks' positions nor the patterns' intensities have significantly changed. This phenomenon results from the material's phase structure remaining mostly unchanged following the alteration.

### 3.4 BET analysis

The specific surface area is a very important measure of the ability to absorb substances. It was used for BET analysis to find out the exact surface area of both natural and modified bentonite. The results showed that the modified bentonite had less surface area than the natural bentonite. It was found that natural bentonite has a surface area of 63.32 m^2^/g. This value went down to 43.31 m^2^/g for modified bentonite. This is because bentonite tends to clump together after being modified by HTAB. Moreover, the organic molecules of the surfactant are stacked on top of the bentonite by entering the voids between its layers. This obstruction impeded the passage between the layers, resulting in a reduction in both the surface area and pore volume [43,44].

### 3.5 Thermogravimetric analyses

**Fig. 6:**
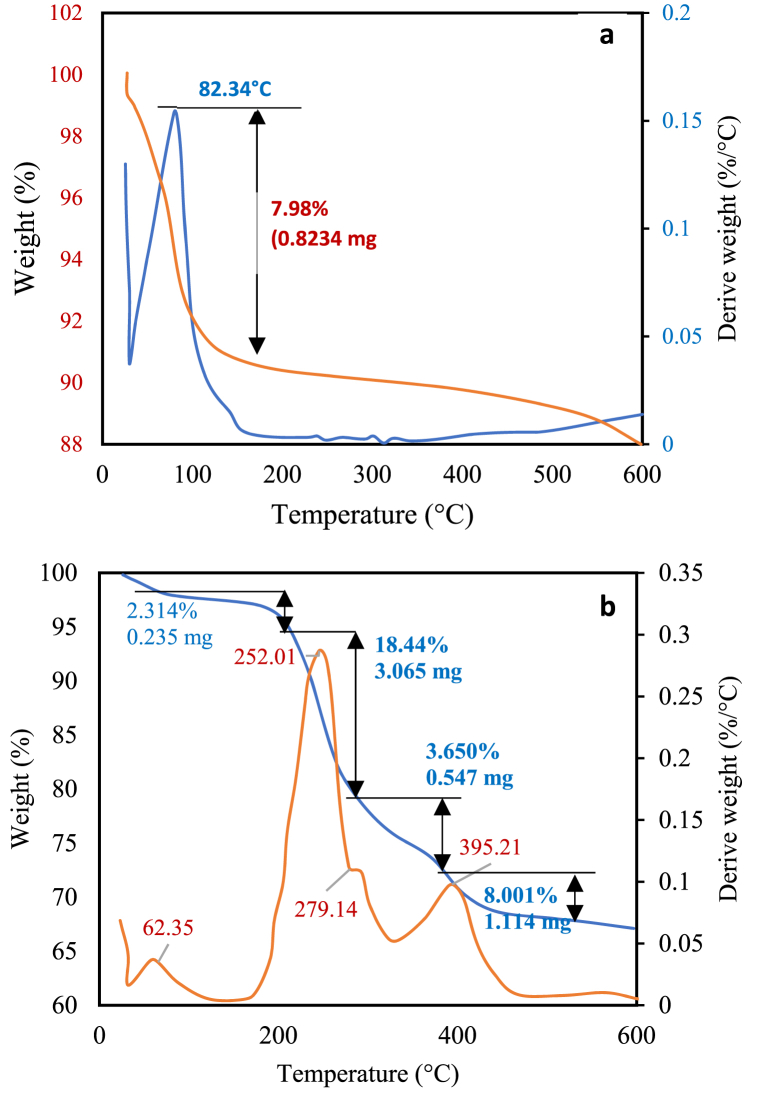
The thermogravimetric TGA/DTG of (a) natural bentonite and (b) modified bentonite.

Fig. 6a and b illustrate the thermal analysis results of natural bentonite and modified bentonite, respectively, using TGA and DTG techniques. Fig. 6a illustrates the thermogravimetric analysis (TGA) and differential thermogravimetry (DTG) curves of the natural bentonite. The graphs depict a solitary phase of mass reduction occurring at an average temperature of 82.34 °C, accounting for 7.98 % of the total mass loss. This reduction is attributed to the release of physically adsorbed water. Conversely, there is no discernible reduction in the quantity of bentonite within the temperature range of 150–500 °C, indicating that the clay exhibits a notable degree of stability within this specific temperature range. Fig. 6b displays the TGA/DTG curves of the modified bentonite. The thermogravimetric analysis (TGA) curve exhibited three distinct phases of mass reduction. During the initial phase, clay dehydration takes place at an average temperature of 62.35 °C, resulting in a mass loss of 2.314 %. The modified bentonite exhibits a lower rate of mass loss compared to natural bentonite, therefore providing evidence for the hydrophobic properties of the modified bentonite [45]. The second stage takes place within the temperature range of 200–325 °C. The DTG analysis showed a distinct peak at 252.01 °C, indicating a mass reduction of 18.44 %. This peak is attributed to the degradation of the surfactant on the bentonite surface, as it is well-established that pure HTAB decomposes at an identical temperature [45]. The third step occurs at temperatures ranging from 325 to 450 °C, leading to a reduction in mass of 8.001 %. This signifies the degradation of the surfactant within the interlayer spaces of the bentonite clay [45,46].

**Fig. 7:**
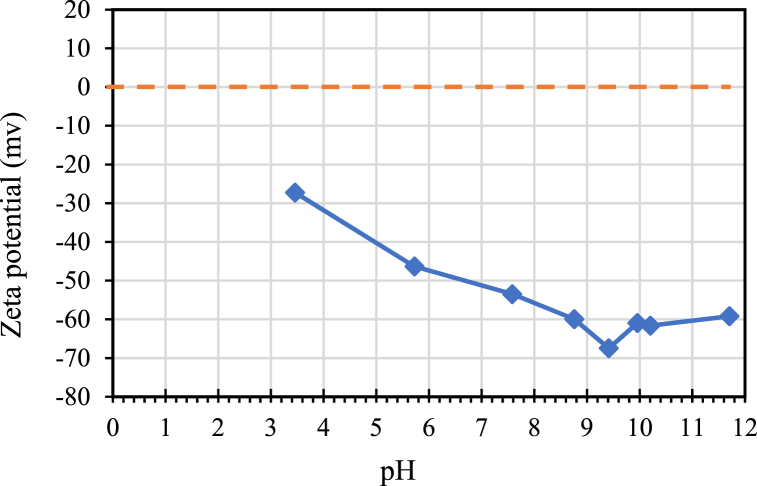
Zetapotential analysis as function of pH for bentonite.

Fig. 7 displays the zeta potentials of the bentonite powders as a function of pH. The isoelectric point (IEP) of bentonite, determined from extrapolation of the experimental data, is around pH 2. Zeta potential levels exceeding 40 mV within the pH range around 9.5 indicate the pH zones where bentonite achieves optimal dispersion. This result is consistent with that of Zhu et al. [47].

### 3.6 Effect of contact time

**Fig. 8:**
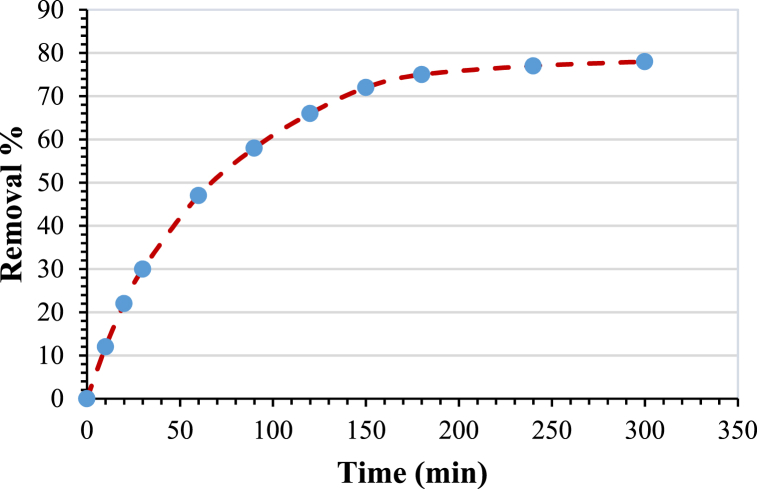
Variation of removal % with time. (Initial concentration C_0_ = 50 ppm, pH = 7, agitation speed = 200 rpm and adsorbent dose = 3 g/L).

The effect of time on the removal of the pollutant was studied using different values, which ranged from 30 to 300 min, while the other parameters were fixed at (the initial concentration of the amoxicillin C_0_ = 50 ppm, pH = 7, agitation speed = 200 rpm, and adsorbent dose = 3 g/L). The influence of contact time on the removal of amoxicillin is shown in Fig. 8. The rate of removal efficiency is fast in the beginning, then decreases slowly until it reaches a constant value (77 % removal) at 240 min. This time is denoted as the equilibrium time at which the quantity of AMX being adsorbed onto the modified bentonite is equal to the AMX desorbed from the adsorbent, and then a dynamic equilibrium is established. This behavior fits with what Gallouze, Halima et al. (2020) [33] found when they looked into how synthetic estrogen could be removed from water by adsorption on modified bentonite.

### 3.7 pH of the solution

**Fig. 9:**
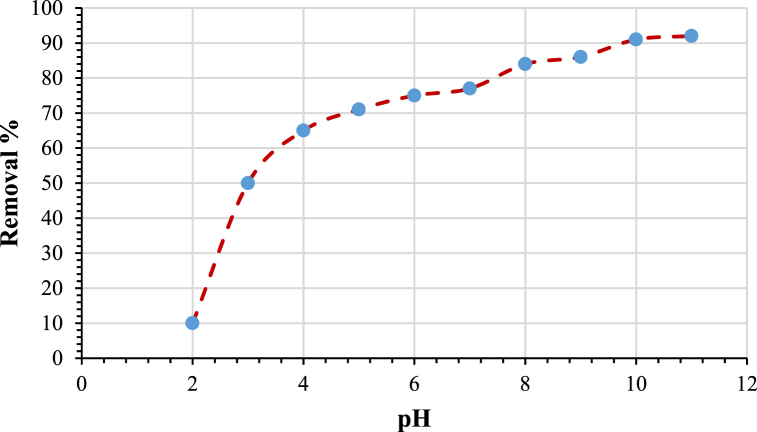
Effect of solution pH on removal percentag. (Initial concentration C_0_ = 50 ppm, Agitation speed = 200 rpm, time = 4 h and adsorbent dose = 3 g/L).

The effect of pH on the adsorption of amoxicillin on modified bentonite was studied experimentally by varying the pH value from 2 to 11 and keeping the other parameters at (C_0_ = 50 ppm, contact time = 4 h, dosage = 3 g/L, and agitation speed = 200 rpm). It is clear from Fig. 9 that adsorption increases with an increase in the pH value and becomes constant at a removal percent value of 91 % after a pH value of 10. This optimum pH value of 10 is used throughout all the adsorption experiments to observe the effect of other parameters like clay dose, mixing time, and agitation speed on adsorption. At acidic pH levels, pH ions are abundant on the adsorption sites of the adsorbent, which compete with the cation groups of the adsorbate, resulting in reduced adsorption capability for cationic organic compounds. With an increase in pH, the surface charge density of H+ diminishes, resulting in a reduction in electrostatic repulsion between the positively charged adsorbent surface and organic adsorbate. Consequently, the degree of adsorption is enhanced [48]. This is in agreement with Zhang et al. (2013) [49], who investigated the removal of methylene blue by multiporous palygorskite modified by ion beam bombardment, as the removal increases with increasing the pH value.

### 3.8 Agitation speed

**Fig. 10:**
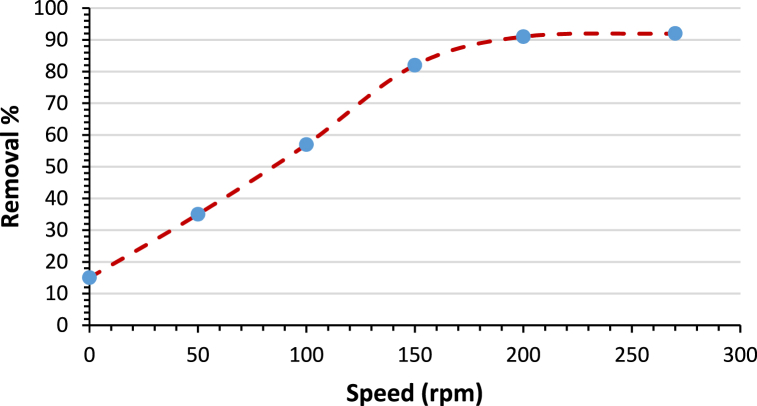
Effect of agitation speed on removal percentage%. (Initial concentration C_0_ = 50 ppm, pH = 10, time = 4 h and adsorbent dose = 3 g/L).

Different agitation speeds (between 0 and 270 rpm) were used in experiments to find out how agitation speed affected the uptake of AMX onto modified bentoniteThe other parameters were kept constant (initial pollutant concentration C_0_ = 50 ppm, pH = 10, time = 4 h, and adsorbent dose = 3 g/L). As shown in Fig. 10, the uptake of AMX was higher at higher values of agitation speed and reached a maximum (91 %) at 200 rpm. Higher values than 200 rpm do not affect the improvement of AMX uptake. This can be attributed to the fact that, at low agitation speeds, the adsorbate faces resistance in the liquid phase through the boundary layer as it moves toward the solid surface of the adsorbent because the film around the solid surface is thicker and the film diffusion seems to be the rate-limiting factor. Increasing the agitation speed will reduce the thickness of the boundary layer, which in turn reduces the resistance to the mass transfer of the adsorbate and hence increases the uptake process [50,51].

### 3.9 Initial pollutant concentration

**Fig. 11:**
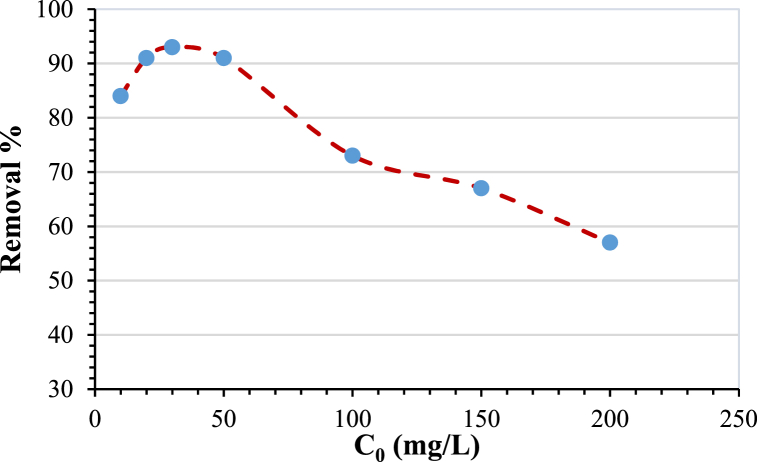
Effect of initial concentration on removal percentage%. (Agitation speed = 200 rpm, pH = 10, time = 4 h and adsorbent dose = 3 g/L).

The initial concentration of AMX has a major impact on the adsorption using modified bentonite. Different values of adsorbate initial concentrations were investigated, ranging from 10 to 200 ppm. The effect of initial concentration on AMX removal is shown in Fig. 11. The removal efficiency increased with increasing the initial concentration in the range of 10–30 ppm, after which the removal efficiency began to decrease and reached 57 % at the initial concentration of 200 ppm. This behavior can be explained by the fact that at low values of initial adsorbate concentration, the ratio of the accessible active sites of the bentonite to the initial number of AMX molecules is high; therefore, the removal efficiency is high. At higher values of initial concentration, more AMX in the solution will surround the active sites of the bentonite, and hence, the remaining vacant adsorption sites of the bentonite will decrease, which will decrease the removal efficiency of the AMX [52].

### 3.10 Modified-bentonite dosage

**Fig. 12:**
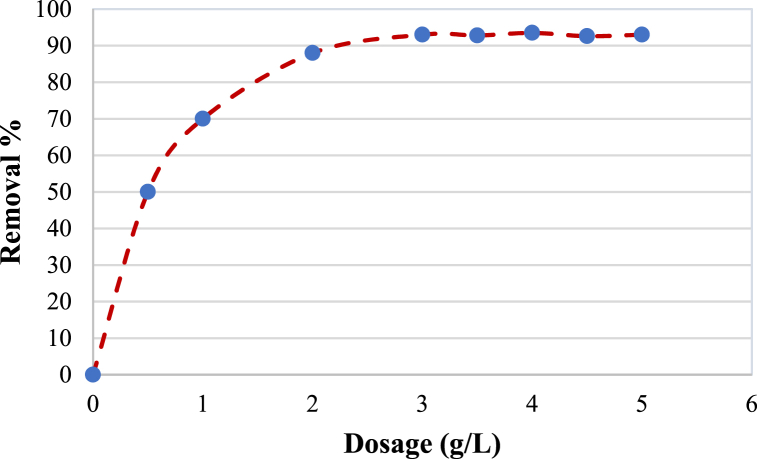
Effect of adsorbent dosage on removal percentage%. (Initial concentration C_0_ = 30 ppm, pH = 10, time = 4 h and agitation speed = 200 rpm).

The effect of the modified bentonite dosage on the adsorption process was studied in the range of 0.2–5 g per 1 L of AMX solution. The other parameters were fixed at (C_0_ = 30 ppm, pH = 10, time = 4 h, and agitation speed = 200 rpm). Fig. 12 shows that with an increase in the dose of modified bentonite, the removal percentage increases and reaches a constant value (93 %) at a modified bentonite dosage of 3 g/L amoxicillin solution. Increasing the adsorbent dosage will increase the active sites of the bentonite, which will adsorb more AMX molecules and hence increase the removal efficiency of the adsorbate. These results worked well with Khan and Hegde (2018) [53], who observed that with an increase in the dose of clay, the adsorption efficiency increases, and this is because of an increase in the surface area of the adsorbent by studying the removal of cadmium from wastewater using modified bentonite.

## 4 Adsorption isotherms

The equilibrium of the adsorption was examined using two adsorption isotherms: Langmuir and Freundlich. The isotherm of Langmuir is based on the assumption of monolayer adsorption; monolayer adsorption occurs at the surface of the adsorbent, which contains a finite number of adsorption sites. Equations (3), (4) represent the mathematical expression of the Langmuir isotherm in the form of non-linear and linear, respectively [54].

(3) $$q_{e}=\frac{K_{L}{\;q}_{m}{\;C}_{e}}{1+K_{L}C_{e}}$$

The linearized form of Eq. (3) is given by Eq. (4)

(4) $$\frac{1}{q_{e}}=\frac{1}{q_{m}}+\frac{1}{q_{m}K_{L}}\;\frac{1}{C_{e}}$$

where.

$q_{e}$: Amount of amoxicillin adsorbed per unit mass of bentonite at equilibrium (mg/g).

$q_{m}$: Maximum capacity of adsorption (mg/g).

$K_{L}$: Constant in Langmuir isotherm (L/mg).
$C_{e}$: The equilibrium concentration of the AMX in the solution at equilibrium (mg/L).

The values of both $K_{L}$ and $q_{m}$ can be calculated using adsorption experimental data and Eq. (4).

The isotherm model of Freundlich is based on the assumption of heterogeneous surface energies and multi-layer adsorption. The Freundlich isotherm is given in the form of non-linear and linear by Eqs. (5), (6), respectively [55].

(5) $$q_{e}=K_{F}C_{e}^{\frac{1}{n}}$$

Eq. (6) represents the linearization form of the Freundlich model

(6) $$\ln\left(q_{e}\right)=\ln\left(K_{F}\right)+\frac{1}{\;n\;}\ln\left(C_{e}\right)$$

The constant parameters of the Freundlich isotherm ($K_{F}$ and n) represent the adsorption capacity and the adsorption intensity, respectively. These values can be predicted from the experimental data using Eq. (6).

**Fig. 13:**
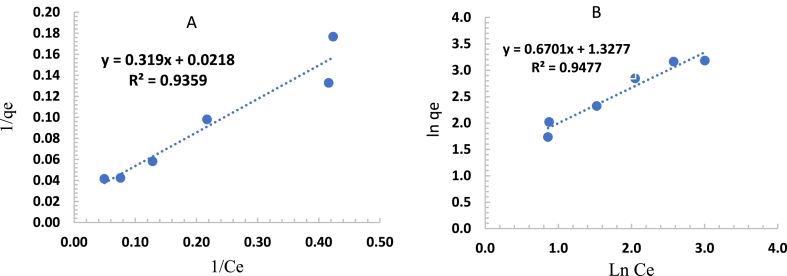
Linear form of the isotherm models for sorption of amoxicillin onto modified-bentonite, (A) Langmuir, (B) Freundlich.

The fitting of experimental data according to Langmuir and Freundlich isotherm models is shown in Fig. 13.

The values of Langmuir isotherm parameters $\left(q_{m},K_{L}\right)$ and Freundlich isotherm $\left(n,K_{F}\right)$, as well as the correlation coefficient ($R^{2})$ are listed in Table 3.

**Table 3:** Parameters of Langmuir and Freundlich equations.

Langmuir			Freundlich		
$K_{L}$	$q_{m}$	$R^{2}$	$K_{F}$	n	$R^{2}$
(L/mg)	(mg/g)				
14.636	3.134	0.9359	3.772	1.492	0.9477

From the values of the correlation coefficients ($R^{2}$), we conclude that the Freundlich model fits the experimental data better than the Langmuir model, which suggests heterogeneity in the multilayer adsorption of amoxicillin onto modified bentonite. In this investigation, we utilized Akaike's information criterion (AIC) [56] to assess the effectiveness of the models, without considering the impact of the number of parameters in the model. The model with the lowest AIC value would be considered the best one to fit the experimental data [57,58]. To assess AIC, it is necessary to know the error sum of squares (SSE). The calculation is derived from the summation of the squared differences between the experimental and predicted values. It can be formulated as:

$$SSE=\sum {\left(q_{e}-q_{e}^{pred}\right)}^{2}$$

AIC is mathematically expressed as:

$$AIC=N\;\ln\left(\frac{SSE}{N}\right)+2K$$

Where N refers to the number of data points and K denotes the number of parameters. The results in Table 4 show that Freundlich's model has lower AIC values.

**Table 4:** Summary of SSE and AIC.

Isotherm model	No of constant	SSE	AIC
Freundlich	2	945.42	31.001
Langmuir	2	2803.18	35.20

**Fig. 14:**
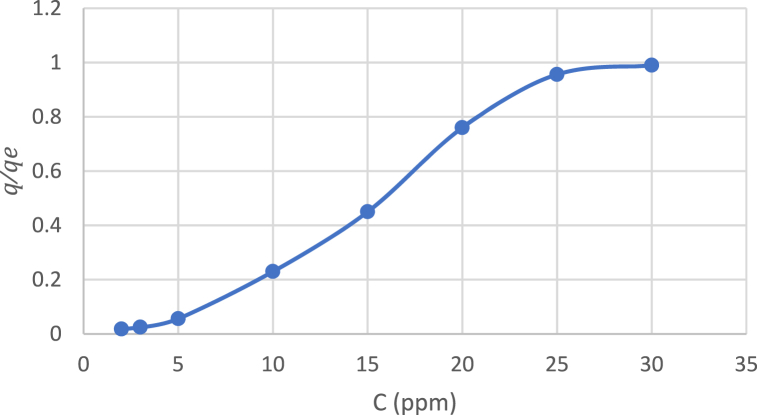
The shape of the adsorption isotherm.

The shape of the adsorption isotherm can be classified as S-shape (S-type), as shown in Fig. 14. The S-shape curve displays a small slope in the beginning, followed by a sharp rise.

### 4.1 Adsorption kinetics

To understand the controlling mechanism and the adsorption rate, the adsorption kinetics of amoxicillin onto modified bentonite were studied using two kinetic models: pseudo-first-order and pseudo-second-order. Eqs. (7), (8) represent the non-linear forms of pseudo-first-order and pseudo-second-order kinetics, respectively [59].

(7) $$q_{t}=\left(\;q_{e}-e^{-k_{1}t}\;\right)$$

(8) $$q_{t}=\frac{k_{2}q_{e}^{2}\;t}{1+k_{2}q_{e}t}$$

The parameters $q_{t}$ and $q_{e}$ (mg/g) represent the amounts of the amoxicillin that adsorbed on the bentonite at any time (t) and equilibrium, respectively. The parameters $K_{1}$ (min^−1^) and $K_{2}\left(g/mg.\;\min\right)$ are the rate constants of the pseudo-first-order and pseudo-second-order kinetics, respectively.

The linearized form of the pseudo-first-order and pseudo-second-order is given by Eqs. (9), (10), respectively [60].

(9) $$\ln\left(q_{e}-q_{t}\right)=\ln\;q_{e}-K_{1}t$$

(10) $$\frac{t}{q_{t}}=\left(\frac{1}{{K_{2}q_{e}}^{2}}\right)+\left(\frac{t}{q_{e}}\right)$$

**Fig. 15:**
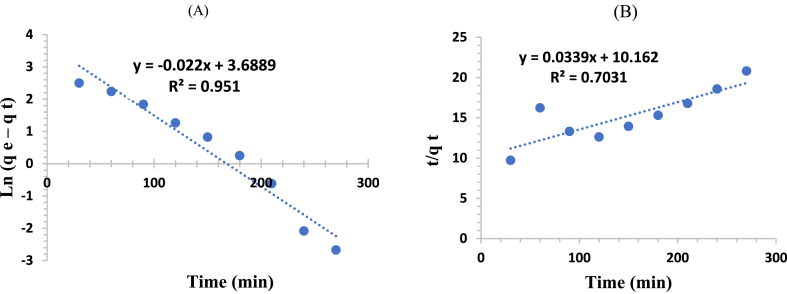
(A) Pseudo-first-order and (B) Pseudo-second-order kinetics models for adsorption of Amoxicillin onto modified-bentonite.

Fig. 15 shows the graphical presentation of the experimental adsorption data according to the pseudo-first-order and the pseudo-second-order kinetics models.

Based on the values of correlation coefficients ($R^{2}$) presented in Table 5, it was concluded that the pseudo-first-order kinetic model describes the experimental data for the adsorption of the AMX onto the modified bentonite.

**Table 5:** Adsorption kinetics parameters of pseudo first-order and pseudo second-order rate equations.

Pseudo-first-order			Pseudo-second-order		
$K_{1}$	$q_{e}$	$R^{2}$	$K_{2}$	$q_{e}$	$R^{2}$
(min^−1^)	(mg/g)		(g/mg. min)	(mg/g)	
0.022	40	0.951	$1.13x10^{-4}$	29.498	0.7031

### 4.2 Thermodynamics study

Three thermodynamic parameters, including enthalpy change $\Delta H^{0}\text{,}$ Gibbs free energy change $\Delta G^{0}$, and entropy change $\Delta S^{0\;}$ were studied to investigate the thermodynamic behavior of the AMX adsorption onto the modified bentonite. The Gibbs free energy change $\Delta G^{0}$ was determined by Eq. (11) [61].

(11) $$\Delta G^{o}=-RTln\left(k_{c}\right)$$

Where R is the universal gas constant (R=8.314kJ/kmol.K), T is the absolute temperature of solution (K), and $K_{c}$ is the distribution coefficient that can be calculated by Eq. (12).

(12) $$K_{c}=\frac{q_{e}}{C_{e}}$$

Where qe is the amount of amoxicillin adsorbed per unit weight of bentonite at equilibrium concentration (mg/g) and Ce is the equilibrium concentration of amoxicillin (mg/L). The relation between the thermodynamic parameters: enthalpy change $\Delta H^{o}$, the entropy change $\Delta S^{o}$ and the Gibbs free energy change $\Delta G^{o}$ is given by Eq. (13) [61].

(13) $$\Delta G^{o}=\Delta H^{o}-T\Delta S^{o}$$

Substitution Eq. (13) in Eq. (11) gives

(14) $$\ln\;K_{c}=\frac{\Delta S{}^{\circ}}{R}-\frac{\Delta H{}^{\circ}}{RT}$$

**Fig. 16:**
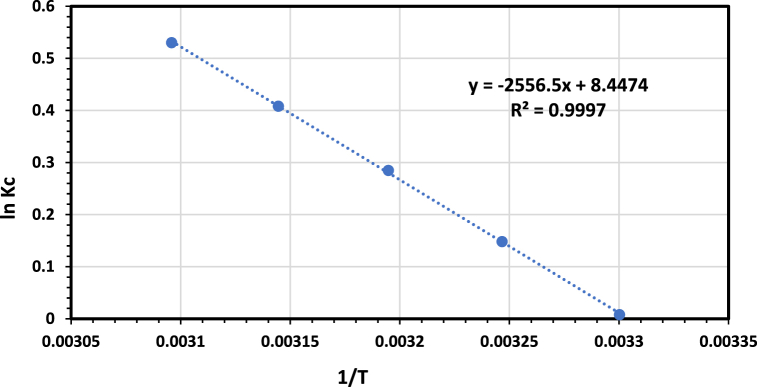
$\ln\;K_{d}$ versus (1/T) for the adsorption of AMX on modified-bentonite.

The values of $\ln\left(K_{c}\right)$ were calculated by Eq. (12) at different temperatures. Plotting $\ln\;\left(K_{c}\right)$ vs $\frac{1}{T}$ gives a linear relation from which the values of $\Delta H^{o}$ and $\Delta S^{o}$ can be calculated. It was found that the adsorption of amoxicillin increased significantly as the temperature changed from 30 to $5{0\;}^{o}C$. Fig. 16 shows that the values $K_{c}$ increase as the temperature increases. Table 6 summarizes the thermodynamic parameters $\Delta G^{o},\Delta H^{o}\;\text{and}\Delta S^{o}$ at various temperatures for the adsorption of amoxicillin onto modified organobentonite.

**Table 6:** Thermodynamic parameters for the adsorption of amoxicillin by the modified-bentonite.

$\Delta G^{o}$ (kJ/mol)			$\Delta S^{o}$ (kJ/mol K)	$\Delta H^{o}$ (kJ/mol)
$3{0\;}^{o}C$	$4{0\;}^{o}C$	$5{0\;}^{o}C$		
−0.01955	−0.74319	−1.42336	0.0702	2.0789

The negative values of $\Delta G^{o}$ indicate the spontaneous nature of the sorption process. The enthalpy change $\Delta H^{o}$ has a positive value, which indicates that the adsorption process is physisorption and endothermic. Physisorption refers to the process of adsorption when the primary molecular interactions between the adsorbate molecules (amoxicillin) and the adsorbent (modified bentonite) are mainly influenced by van der Waals forces. Physisorption is considered to be a weak and reversible phenomenon controlled by the competing actions of adsorption and desorption. These processes occur at varying speeds on the heterogeneous microsurface of the materials. Increasing the temperature will enhance the adsorption process. The positive sign of the entropy shift indicates that the level of disorder at the solid-liquid interface would rise during the adsorption of amoxicillin [62].

## 5 Conclusion

Amoxicillin (AMX) was found to be effectively removed from an aqueous solution by the modified bentonite, with a maximum removal of 93 %. It was found that the pH of the solution, the contact time, the initial concentration, the agitation speed, and the modified bentonite dose all affected the percentage of AMX elimination. The optimal settings for achieving the maximum clearance rate in this study were determined to be 240 min of reaction time, a pH of 10, a stirring speed of 200 revolutions per minute, and a concentration of 1.5 g/L. According to the isotherm analysis, the sorption data and Freundlich had a good correlation (R^2^ = 0.9477) when compared to the Langmuir isotherm model. The experimental data obeyed the pseudo-first-order rather than the pseudo-second-order, as revealed by the kinetic research, showing that physisorption has been the dominant process (R^2^ = 0.997). The thermodynamic analysis revealed that the adsorption process exhibited endothermic characteristics and was found to be spontaneous.

## Sources of financing format

This study did not receive any further support from governmental, corporate, or nonprofit entities.

## Data availability

Data will be made available on request.

## CRediT authorship contribution statement

**Alaa K. Mohammed:** Writing – review & editing, Supervision, Methodology. **Sara M. Saadoon:** Writing – original draft, Methodology, Investigation. **Ziad T. Abd Ali:** Visualization, Supervision, Data curation. **Israa M. Rashid:** Software, Project administration, Conceptualization. **Nadya Hussin AL Sbani:** Validation, Resources.

## Declaration of competing interest

The authors declare that they have no known competing financial interests or personal relationships that could have appeared to influence the work reported in this paper.
